# Supplementary material for: Tephrosin Suppresses the Chemoresistance of Paclitaxel-Resistant Ovarian Cancer via Inhibition of FGFR1 Signaling Pathway
Source: Biomedicines. 2023 Nov 27;11(12):3155. doi: 10.3390/biomedicines11123155 (PMC10740824; doi:10.3390/biomedicines11123155)
Supplement: Supplementary file 1 [file biomedicines-11-03155-s001.zip › Figure S1.pdf]

Figure S1. Tephrosin inhibited the FGFR1/FRS2 signaling pathway, not FGFR2.

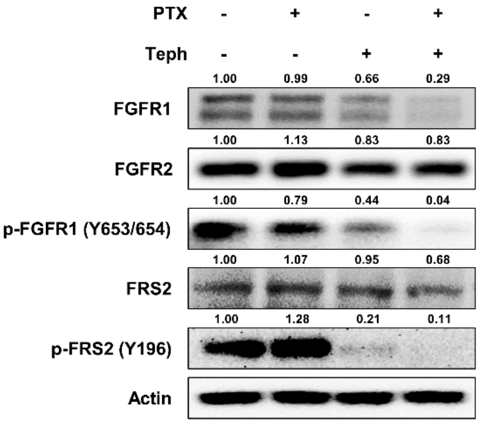

Figure S1. Cells (6.0 x 10^5) were treated with DMSO (mock), paclitaxel (200 nM), tephrosin (10 µM), and combination treatment composed of paclitaxel and tephrosin for 48 h. Immunoblotting was performed to determine the expressions of FGFR1, FGFR2, FRS2, phosphorylation of FGFR1 at Y653/654 residue and phosphorylation of FRS2 at Y196 residue. Actin was used as a loading control. Protein expression levels were quantified relative to the control, following normalization to the corresponding expression of actin using ImageJ software. PTX, paclitaxel; Teph, tephrosin
