# Supplementary material for: Tephrosin Suppresses the Chemoresistance of Paclitaxel-Resistant Ovarian Cancer via Inhibition of FGFR1 Signaling Pathway
Source: Biomedicines. 2023 Nov 27;11(12):3155. doi: 10.3390/biomedicines11123155 (PMC10740824; doi:10.3390/biomedicines11123155)
Supplement: Supplementary file 1 [file biomedicines-11-03155-s001.zip › Figure S2.pdf]

Figure S2. Comparison of P-glycoprotein expression between SKOV3 cells and SKOV3-TR cells

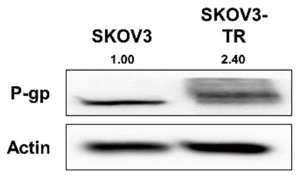

Figure S2. SKOV3 cells were treated with DMSO (mock), and SKOV3-TR cells were treated with paclitaxel (50 nM). After harvesting all the cells, immunoblotting was examined to identify the expression of P-glycoprotein. Actin was used as a loading control. Protein expression levels were quantified relative to the control, following normalization to the corresponding expression of actin using ImageJ software. P-glycoprotein, P-gp
