# Supplementary material for: Tephrosin Suppresses the Chemoresistance of Paclitaxel-Resistant Ovarian Cancer via Inhibition of FGFR1 Signaling Pathway
Source: Biomedicines. 2023 Nov 27;11(12):3155. doi: 10.3390/biomedicines11123155 (PMC10740824; doi:10.3390/biomedicines11123155)
Supplement: Supplementary file 1 [file biomedicines-11-03155-s001.zip › Table S1.pdf]

Table S1. List of primer for RT-PCR and RT-qPCR analysis

Targets	Sequence of primer
FGFR1	F_ 5'- TGGCCCGAGGCATGGAGTAT -3'
	R_ 5'- TCACTCTGGTGGGTGTAGAT -3'
FRS2	F_ 5'- CCATCTTTGCCTCCTGTTTGG -3'
	R_ 5'- TGCCTGTGTTCTAAACTTGGGC -3'
BCL-2	F_ 5'- GGCAATGTGACTTTTTCCAA -3'
	R_ 5'- GGCTGATATTCTGCAACACTG -3'
BCL-XL	F_ 5'- GTAAACTGGGGTCGCATTGT -3'
	R_ 5'- TGGATCCAAGGCTCTAGGTG -3'
MCL-1	F_ 5'- CTCCCCACCAAGAGTCCACA -3'
	R_ 5'- CCCAAACCACTTGGGGTGTC -3'
BAX	F_ 5'- CCAGCTGCCTTGGACTGT -3'
	R_ 5'- ACCCCCTCAAGACCACTCTT -3'
XIAP	F_ 5'- TGGCAGATTATGAAGCACGGATC -3'
	R_ 5'- AGTTAGCCCTCCTCCACAGTGA -3'
SURVIVIN	F_ 5'- CCCATAGAGGAACATAAAAAGCAT-3'
	R_ 5'- TCAAAAATTCACCAAGGGTTAATT-3'
GAPDH	F_ 5'- GTCTCCTCTGACTTCACAGCG -3'
	R_ 5'- ACCACCCTGTTGCTGTAGCCAA -3'
